# Supplementary material for: Fast, efficient, and accurate neuro-imaging denoising via supervised deep learning
Source: Nat Commun. 2022 Sep 2;13:5165. doi: 10.1038/s41467-022-32886-w (PMC9440141; doi:10.1038/s41467-022-32886-w)
Supplement: Supplementary file 1 — Supplementary Information [file 41467_2022_32886_MOESM1_ESM.pdf]

Supplementary Figures

Title

Fast, Efficient, and Accurate Neuro-Imaging Denoising via Supervised Deep Learning

Author List

Shivesh Chaudhary[1], Sihoon Moon[1], Hang Lu[1,2,*]

Affiliations

[1]School of Chemical & Biomolecular Engineering, Georgia Institute of Technology, Atlanta, Georgia, United States of America

[2]Petit Institute for Bioengineering and Bioscience, Georgia Institute of Technology, Atlanta, Georgia, United States of America

[*]Correspondence should be addressed to HL: hang.lu@gatech.edu

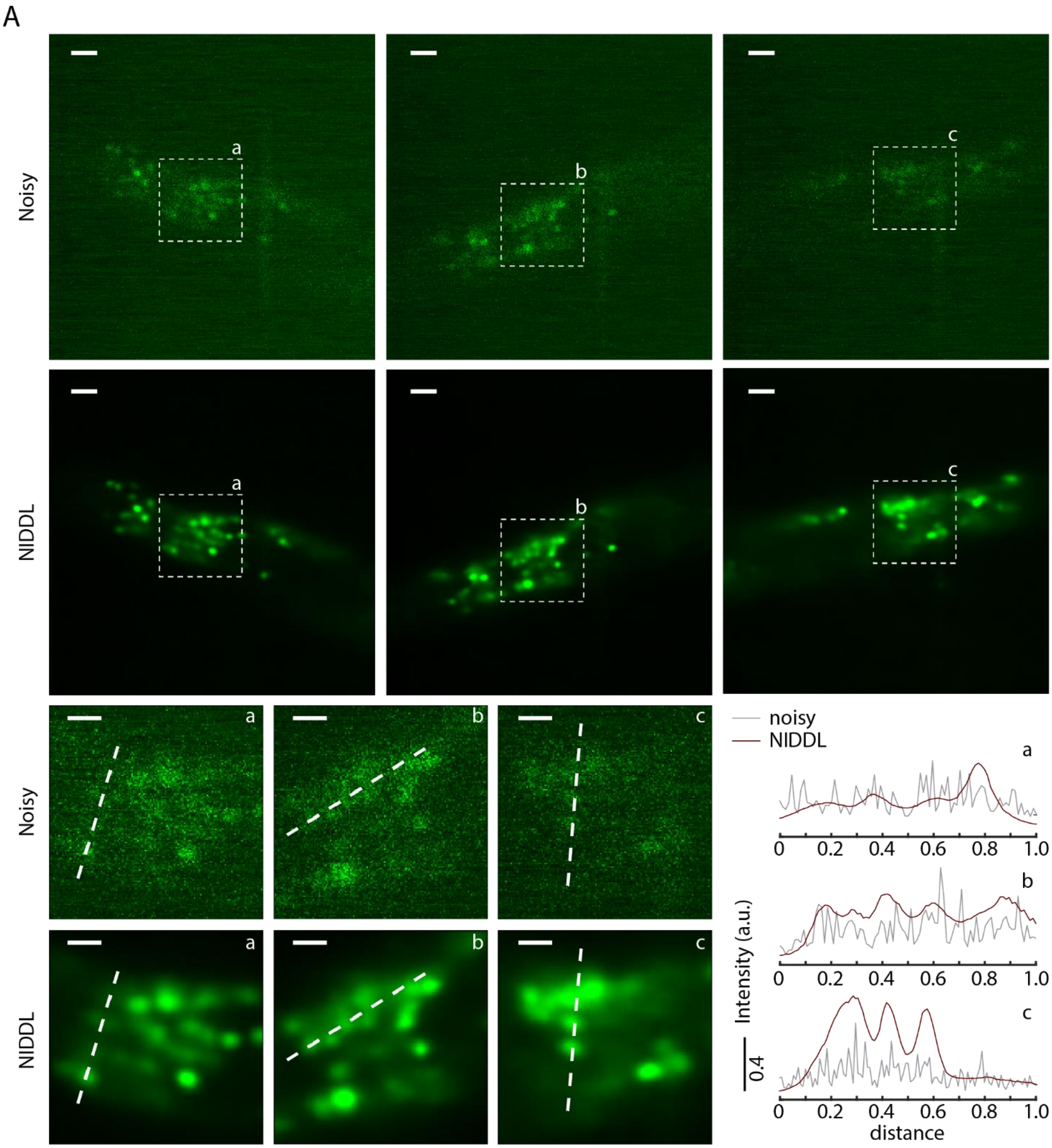

Supplementary Figure 1. Additional examples of whole-brain noisy images denoised by trained network. A) Top – random example noisy images (acquired at low laser power) from different animals (1 z-plane from 3D image stack) and corresponding denoised output, (scale bar - 10 µm). Bottom left – zoom ins of image portions highlighted with dotted box in top panel, (scale bar - 5 µm). Bottom right – pixel intensities extracted from noisy images and deep denoised images along the dotted lines shown in insets. Clear peaks and valleys in intensity profiles in denoised images correspond to individual nuclei. Data come from strain ZIM504.

A

Unet

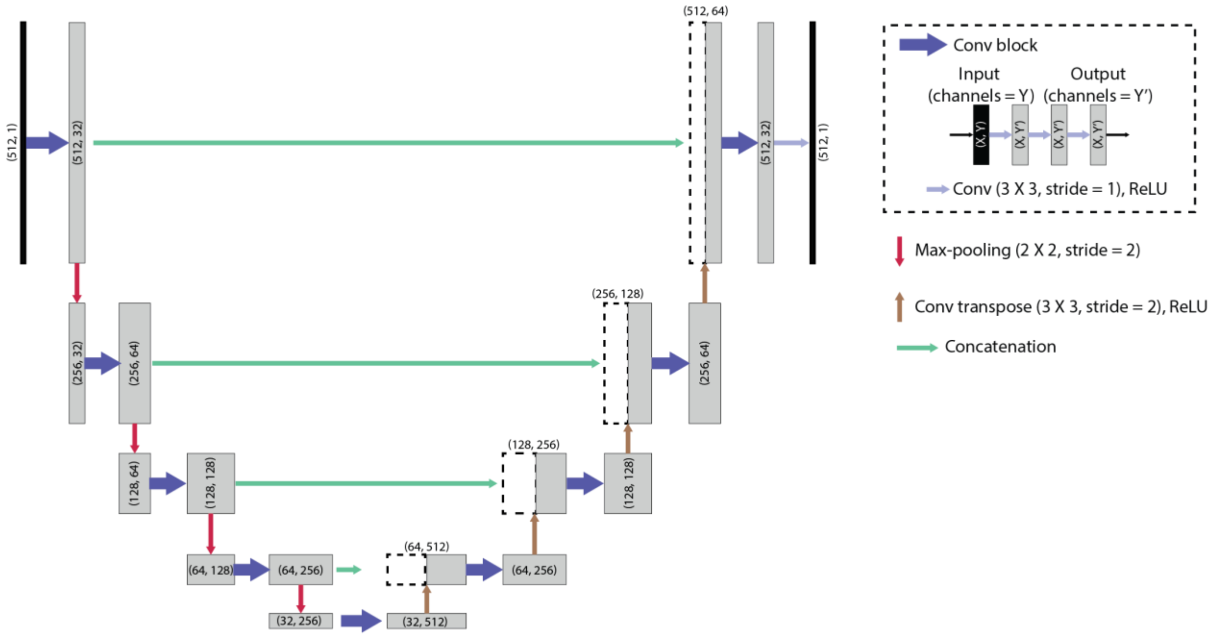

B

Unet_fixed

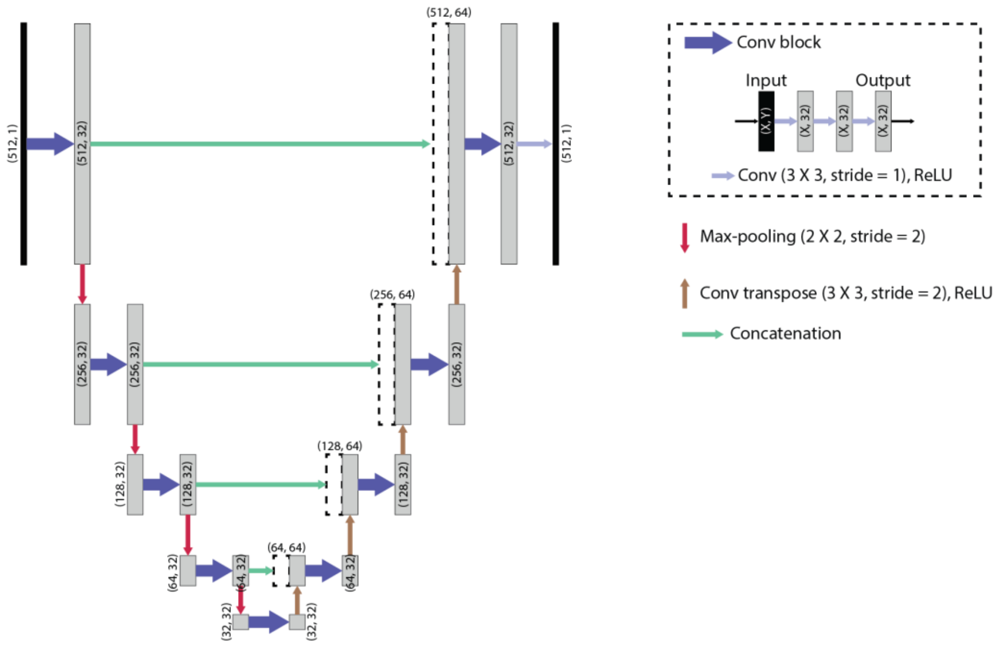

Supplementary Figure 2: Architecture details of variants of UNets tried for denoising images. A) Traditional UNet architecture. B) Optimized UNet architecture with fixed channel depth.

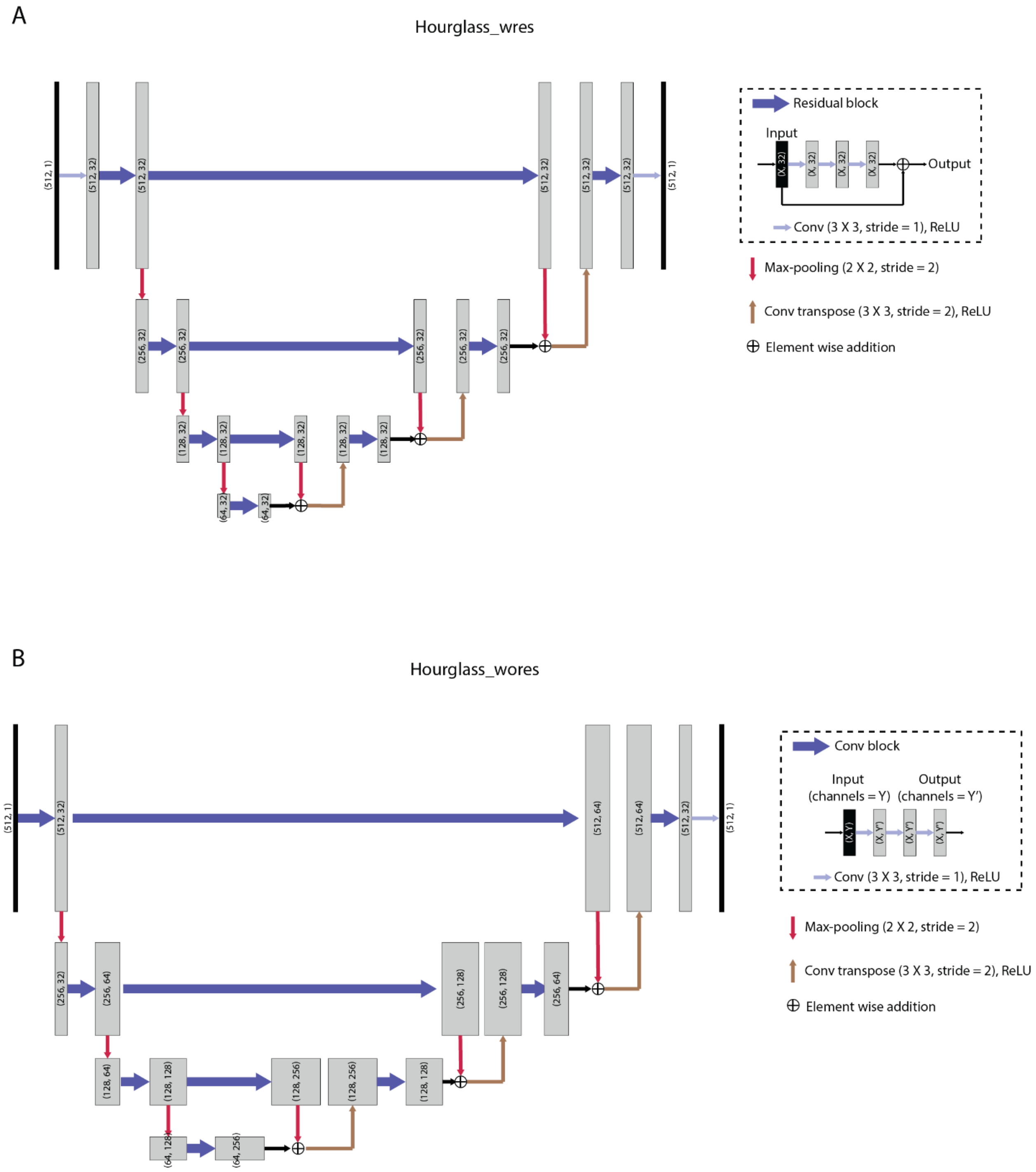

Supplementary Figure 3: Architecture details of variants of Hourglass architecture tried for denoising images. A) Optimized hourglass architecture with fixed channel depth and residual connections in convolution block (conv block). B) Hourglass architecture with channel depth doubling after max-pooling operations and no residual connections in conv block.

A

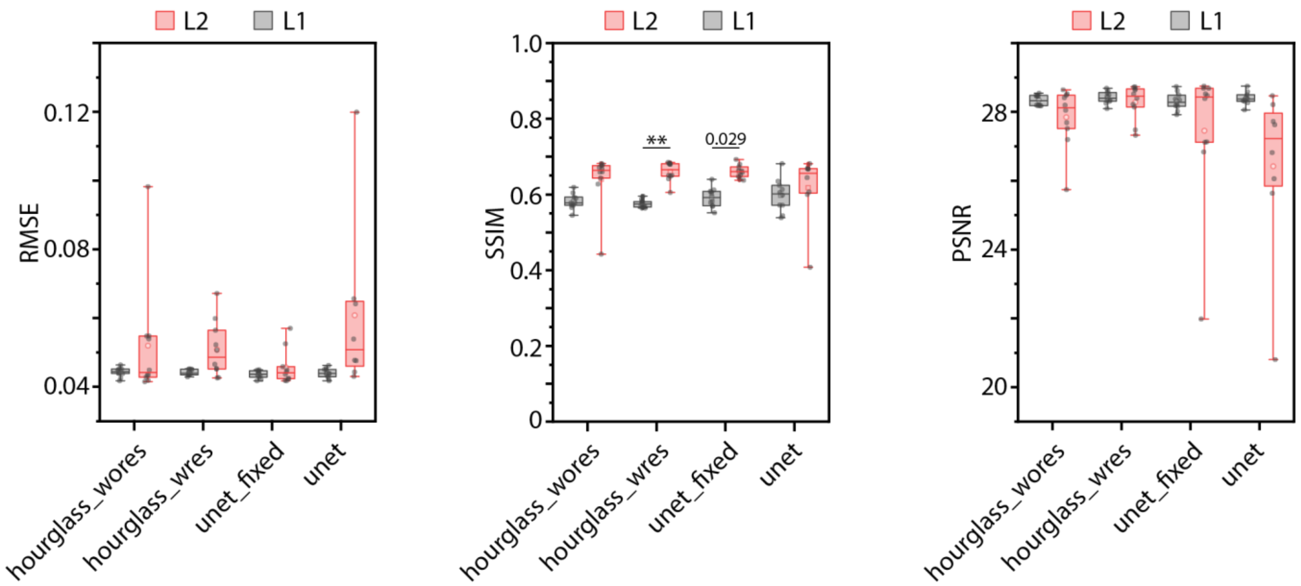

Supplementary Figure 4. Denoising accuracy comparison across neural network architecture variants and loss functions. A) Left - RMSE accuracy, middle – SSIM accuracy and right - PSNR of denoised images across architectures trained with specific loss functions (L1 and L2 loss). Each dot in every panel corresponds to mean accuracy on 600 test images for one instance of trained network. In total, 10 instances were trained for each condition with random subset of total data used for training each instance. Data come from strain ZIM504. (n = 10, **p<0.01, two-sided Holm-Bonferroni paired comparison test). Box center indicates median, box edges indicate 25th and 75th percentile, and whiskers indicate 5th and 95th percentile. Source data are provided in Source Data file.

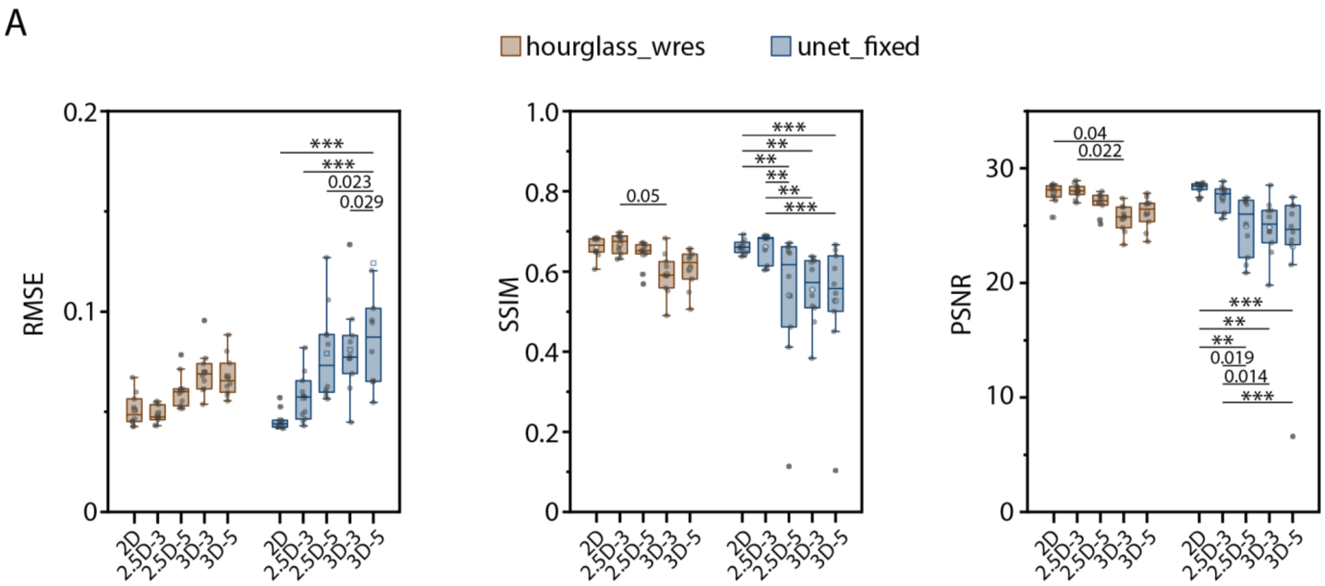

Supplementary Figure 5. Denoising accuracy comparison across training modes for the optimized architectures 'unet_fixed' and 'hourglass_wres'. See Methods – Network Optimization 3 for description of training modes. A) Left - RMSE accuracy, middle – SSIM accuracy and right - PSNR of denoised images across training modes for 'hourglass_wres' and 'unet_fixed' architectures. Each dot in every panel corresponds to mean accuracy on 600 test images for one instance of trained network. In total, 10 instances were trained for each condition with random subset of total data used for training each instance. Data come from strain ZIM504. (n = 10, ***p<0.001, **p<0.01, two-sided Holm-Bonferroni paired comparison test). Box center indicates median, box edges indicate 25th and 75th percentile, and whiskers indicate 5th and 95th percentile. Source data are provided in Source Data file.

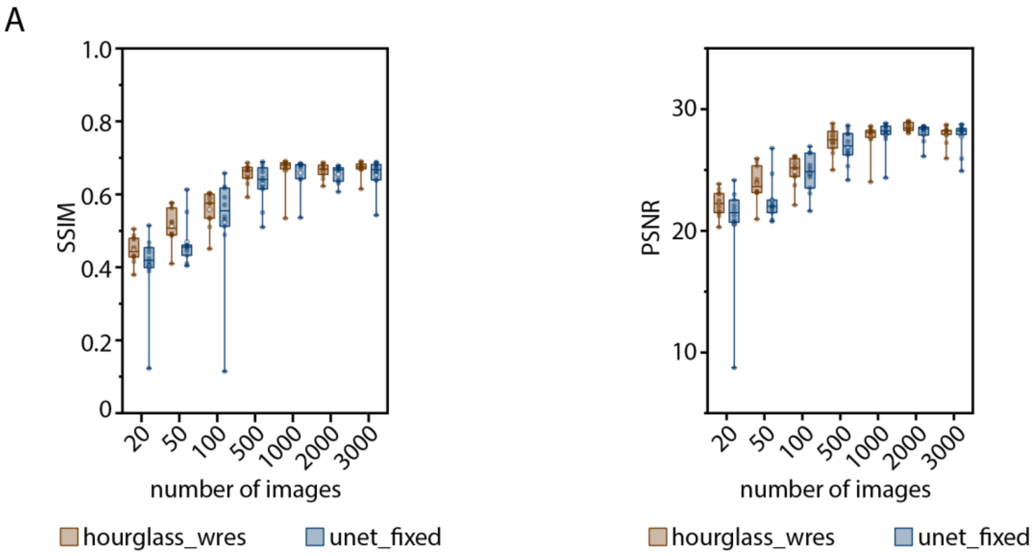

Supplementary Figure 6. Accuracy vs training data size trade-off for optimized architectures. A) Left – SSIM accuracy, right – PSNR of denoised images vs number of images used for training the networks. Each dot corresponds to mean accuracy on 600 test images for one instance of trained network. In total, 10 instances were trained for each condition with random condition specific subset of total data used for each training instance. Data come from strain ZIM504. Box center indicates median, box edges indicate 25th and 75th percentile, and whiskers indicate 5th and 95th percentile. Source data are provided in Source Data file.

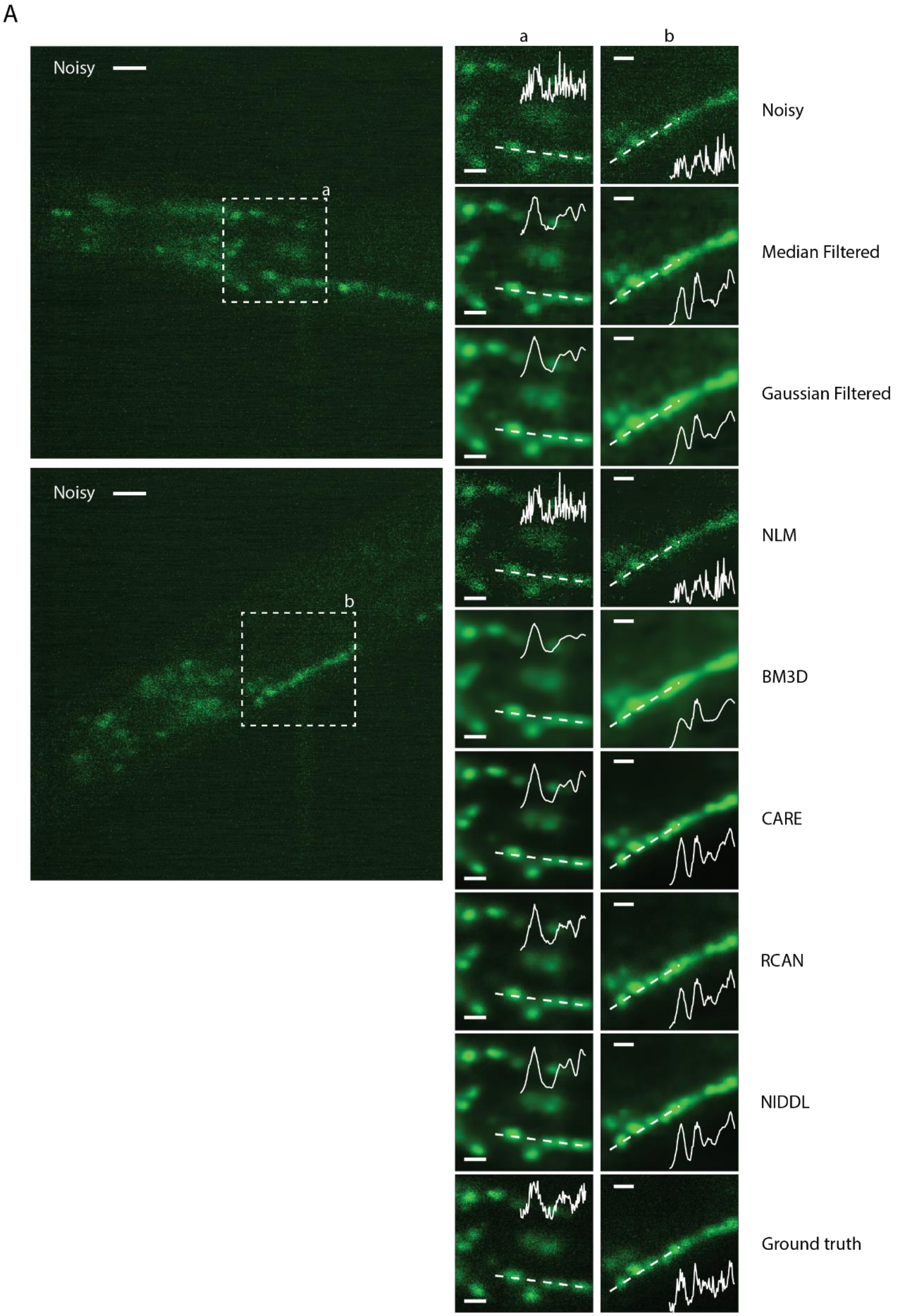

Supplementary Figure 7. Additional qualitative comparison examples of denoising methods on whole-brain images. Left - example noisy images (single z planes) from noisy whole-brain image stacks

(acquired at low laser power) (scale bar - 10 µm). Right – corresponding denoised output generated by different methods shown for dotted boxes in noisy images. (scale bar - 5 µm). Cell nuclei are labelled with nuclear localized GCaMP5K. Data come from strain ZIM504. Inset shows intensity profile along the dotted line.

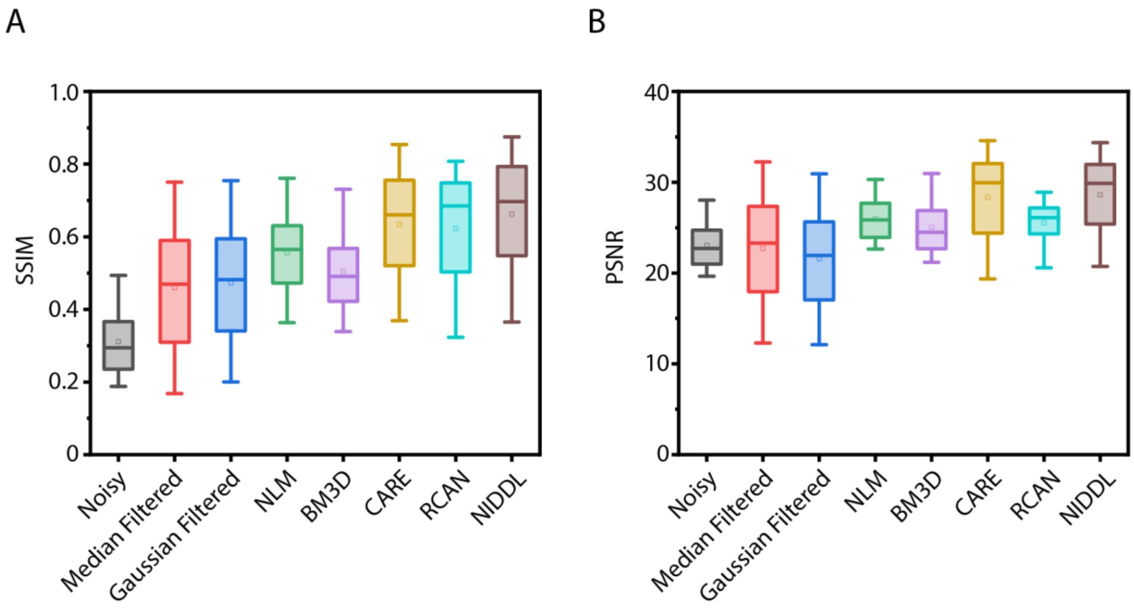

Supplementary Figure 8. Additional accuracy comparison of various denoising methods for whole-brain images. A) SSIM accuracy, B) PSNR across noisy images, median filtered images, Gaussian filtered images, and deep denoised images (n = 600 images). Data come from strain ZIM504. Box center indicates median, box edges indicate 25th and 75th percentile, and whiskers indicate 5th and 95th percentile. Source data are provided in Source Data file.

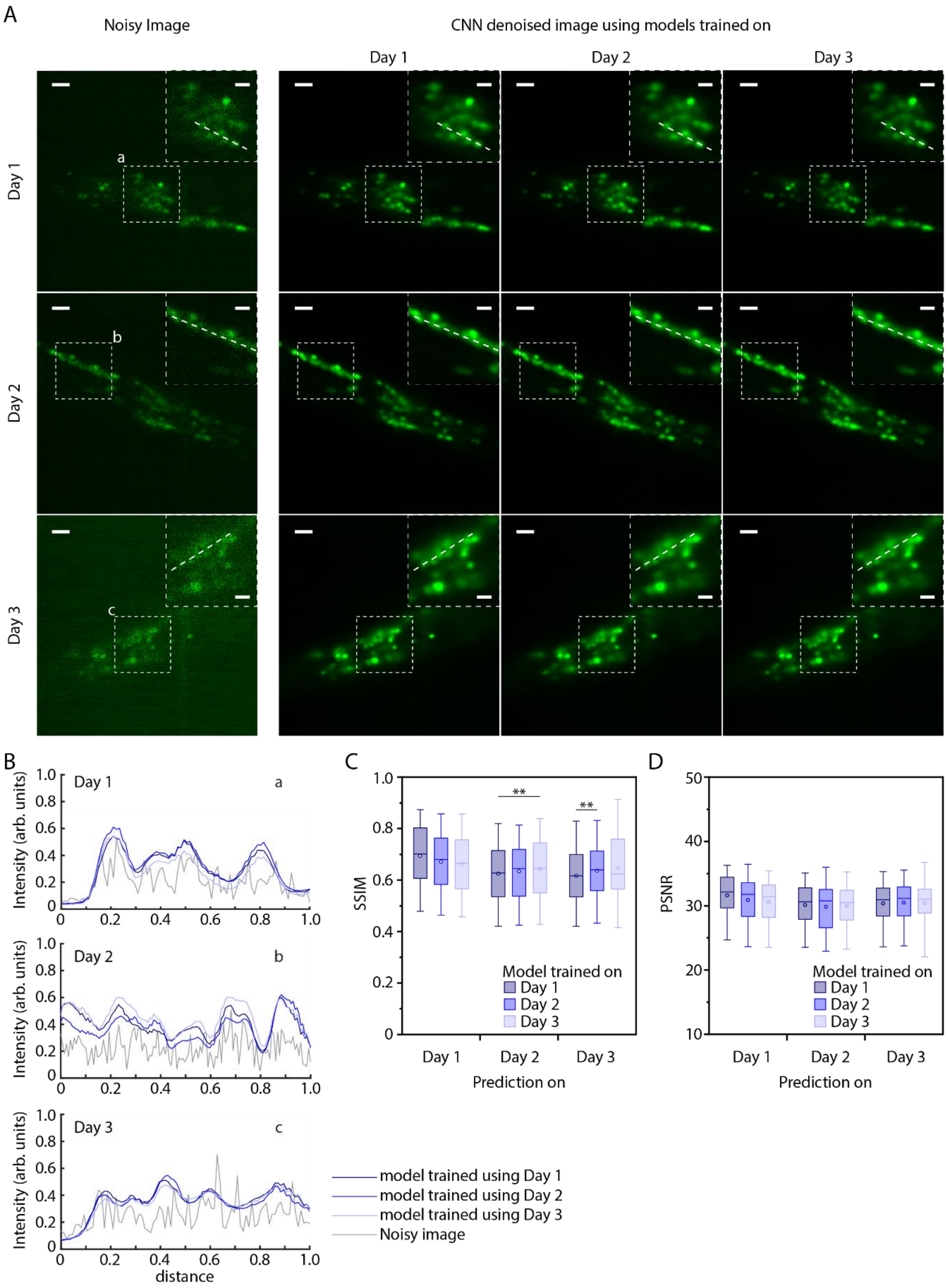

Supplementary Figure 9. Additional deep denoising accuracy quantification on images from 3 different imaging sessions (different days) when model is trained with specific day's data only. Data come from ZIM504 strain. A) Left – example noisy images from 3 imaging sessions, right – corresponding denoised

image outputs by 3 different networks trained on specific day's data. (scale bar - 10 μm). Inset images show zoom-ins of dotted boxes (scale bar - 5 μm). B) Intensity profiles along the dotted lines shown in insets in A for noisy images and denoised images output by 3 different networks. C) SSIM accuracy and D) PSNR achieved by deep denoising on noisy images from 3 imaging sessions when networks are trained on specific strain's data. (n = 40, 1,043, 1,043, 1,532, 50, 1,532, 1,403, 1,403, 50 images for 9 conditions, **p<0.01, two-sided Holm-Bonferroni paired comparison test). Box center indicates median, box edges indicate 25th and 75th percentile, and whiskers indicate 5th and 95th percentile. Source data are provided in Source Data file.

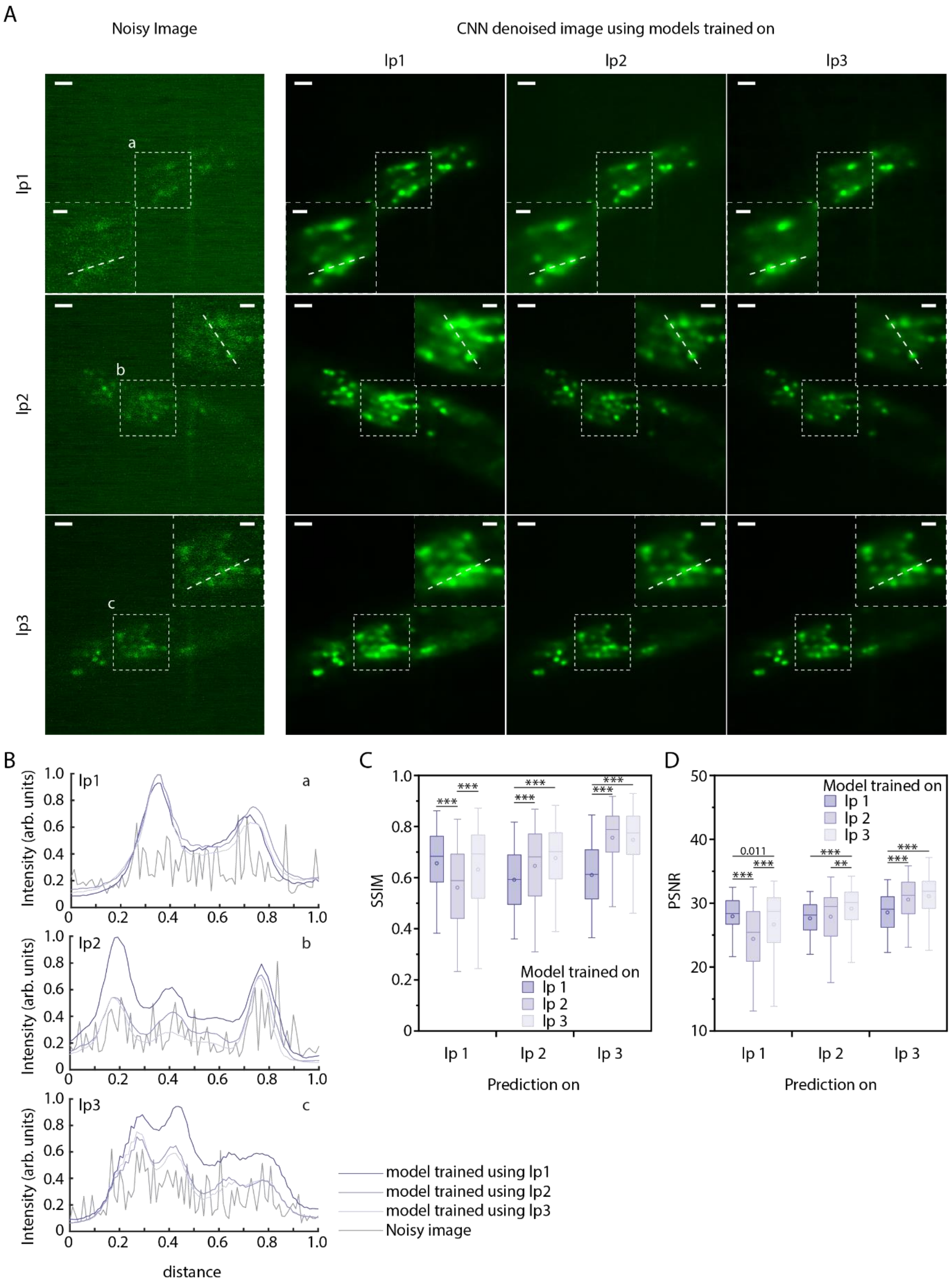

Supplementary Figure 10. Additional deep denoising accuracy quantification on images acquired with 3 different low laser power settings when model is trained with specific setting's data only. Data come from ZIM504 strain. A) Left – example noisy images acquired across 3 laser power settings, right –

corresponding denoised image outputs by 3 different networks trained on specific settings' data. (scale bar - 10 µm). Inset images show zoom-ins of dotted boxes (scale bar - 5 µm). B) Intensity profiles along the dotted lines shown in insets in A for noisy images and denoised images output by 3 different networks. C) SSIM accuracy and D) PSNR achieved by deep denoising on noisy images across 3 laser power settings when networks are trained on specific setting's data. ((n = 88, 2,728, 2,728, 2,262, 100, 2,262, 2,806, 2,806, 226 images for 9 conditions, ***$p<0.001$, **$p<0.01$, two-sided Holm-Bonferroni paired comparison test). Box center indicates median, box edges indicate 25th and 75th percentile, and whiskers indicate 5th and 95th percentile. Source data are provided in Source Data file.

A

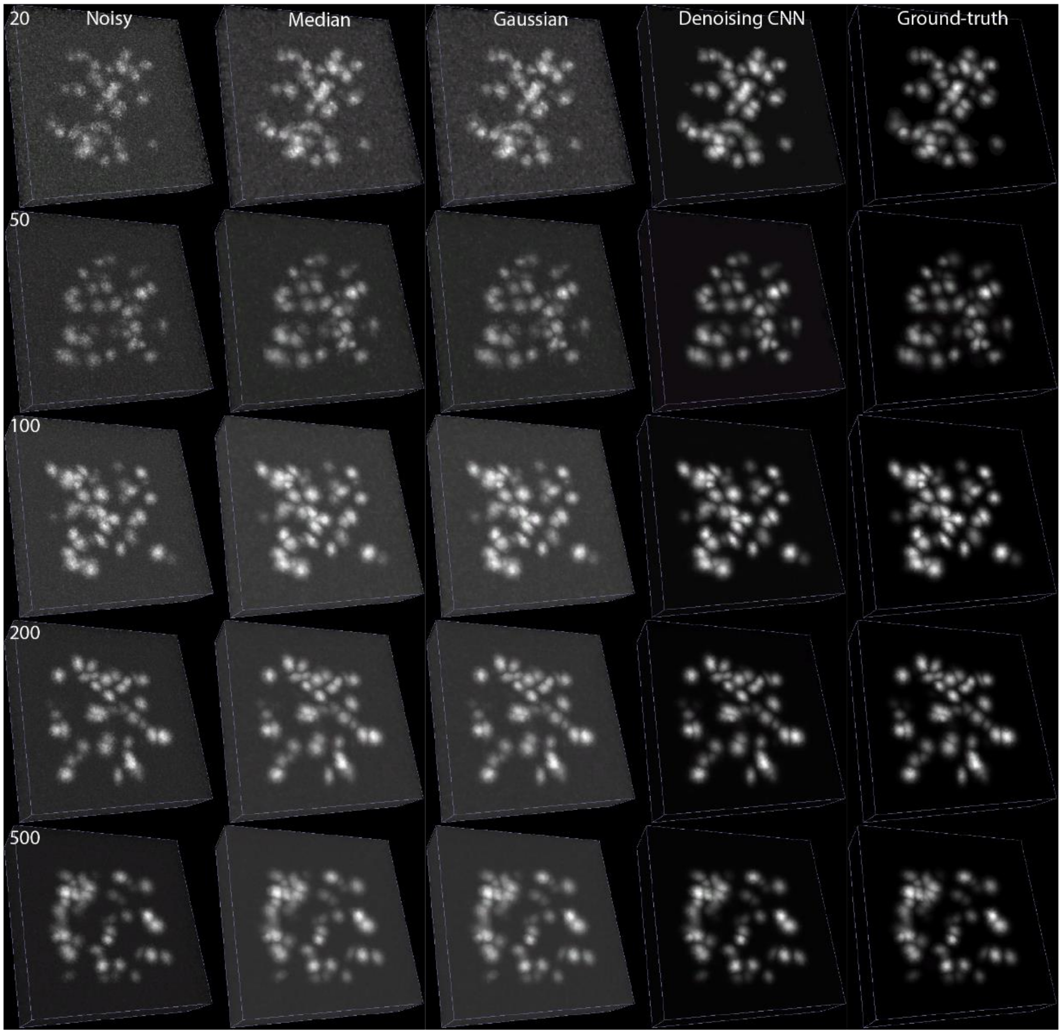

B

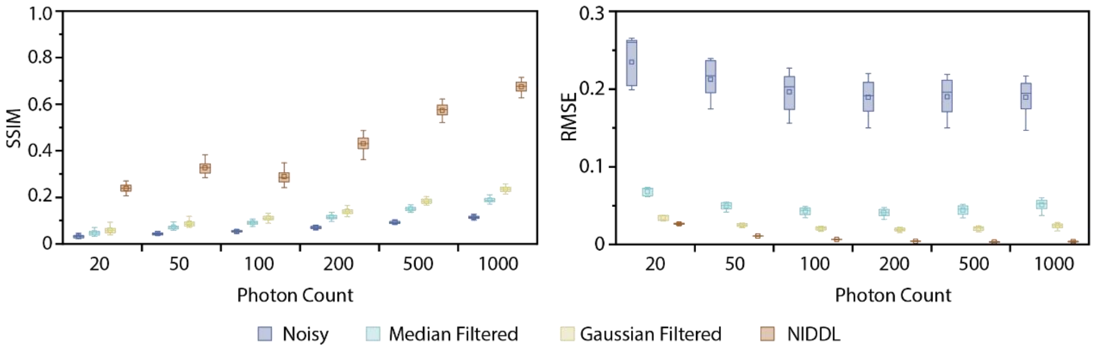

Supplementary Figure 11. Additional denoising accuracy quantification on synthetic images. A) Examples of synthetic noisy and ground-truth image stacks across range of signal levels in images (photon count levels, see Methods - synthetic image data generation for details), and corresponding denoised images generated by median filtering, Gaussian filtering, deep denoising. B) Left – SSIM, Right - RMSE comparison across methods on synthetic images across range of photon count levels (n = 100 image stacks). Box center indicates median, box edges indicate 25th and 75th percentile, and whiskers indicate 5th and 95th percentile. Source data are provided in Source Data file.

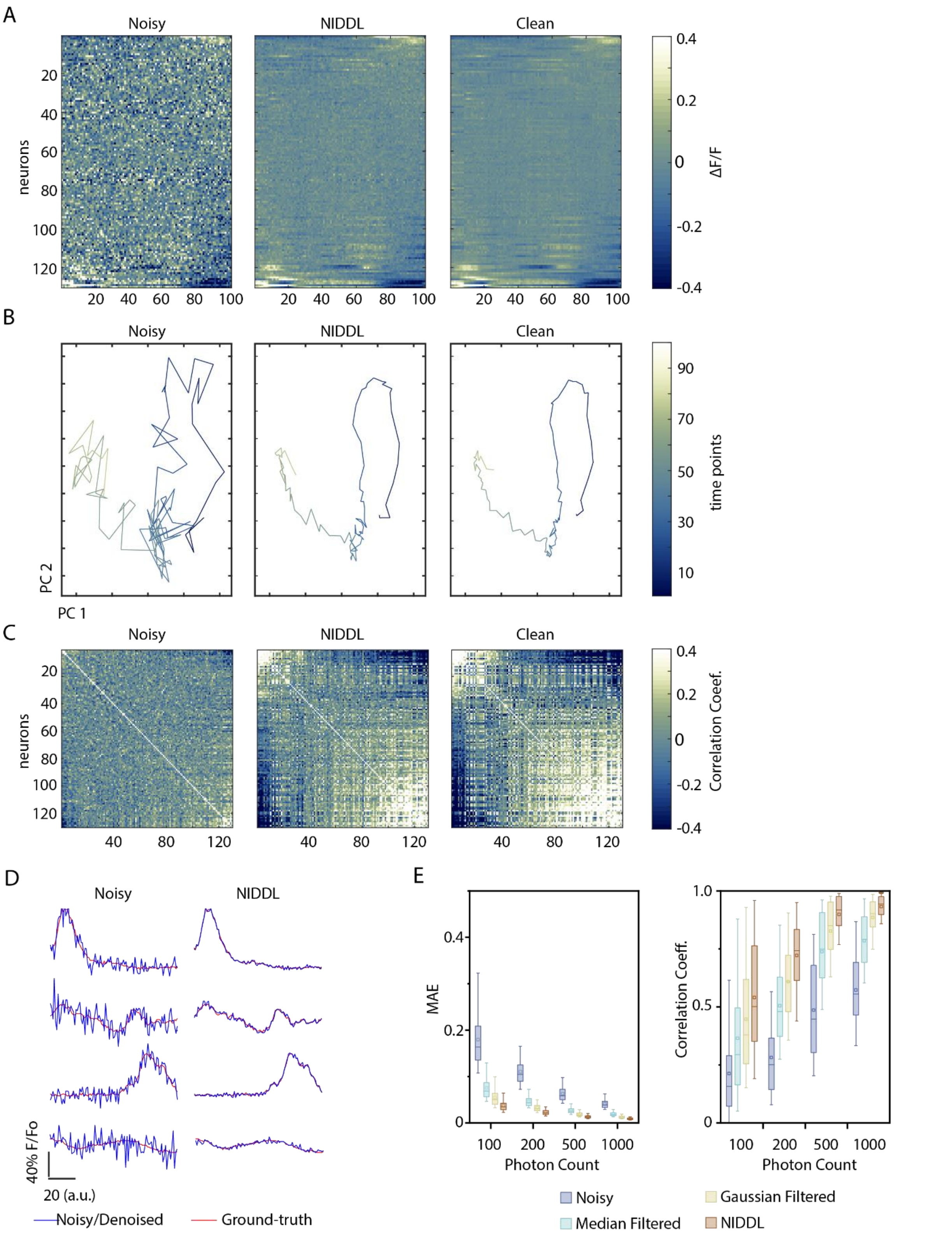

Supplementary Figure 12. Accuracy quantification of traces extracted from deep denoised videos using semi-synthetic whole-brain video datasets (see Methods – Semi synthetic video data generation). A) Heatmap showing neuron activity traces of cells in a semi-synthetic noisy video, corresponding ground-truth video, and traces extracted from video denoised with deep network. (Video corresponds to 200

photon count level). B) Neuron activities from noisy video, ground-truth video and deep denoised video in A projected on to first 2 PC's. Deep denoising recovers low-dimensional neuron activity dynamics. C) Pairwise Pearson correlation across neurons of neuron activity traces extracted from Noisy, NIDDL denoised and ground-truth video. D) Comparison of traces in noisy video to traces in ground-truth video for few example cells before and after NIDDL denoising (same video as in A). E) Left – mean absolute error (MAE) of traces to ground-truth traces, right – Pearson correlation coefficient of traces to ground-truth traces across denoising methods and signal levels on videos (photon count levels). (N = 1 video, n = 130 cells in each video for each condition). Box center indicates median, box edges indicate 25th and 75th percentile, and whiskers indicate 5th and 95th percentile. Source data are provided in Source Data file.

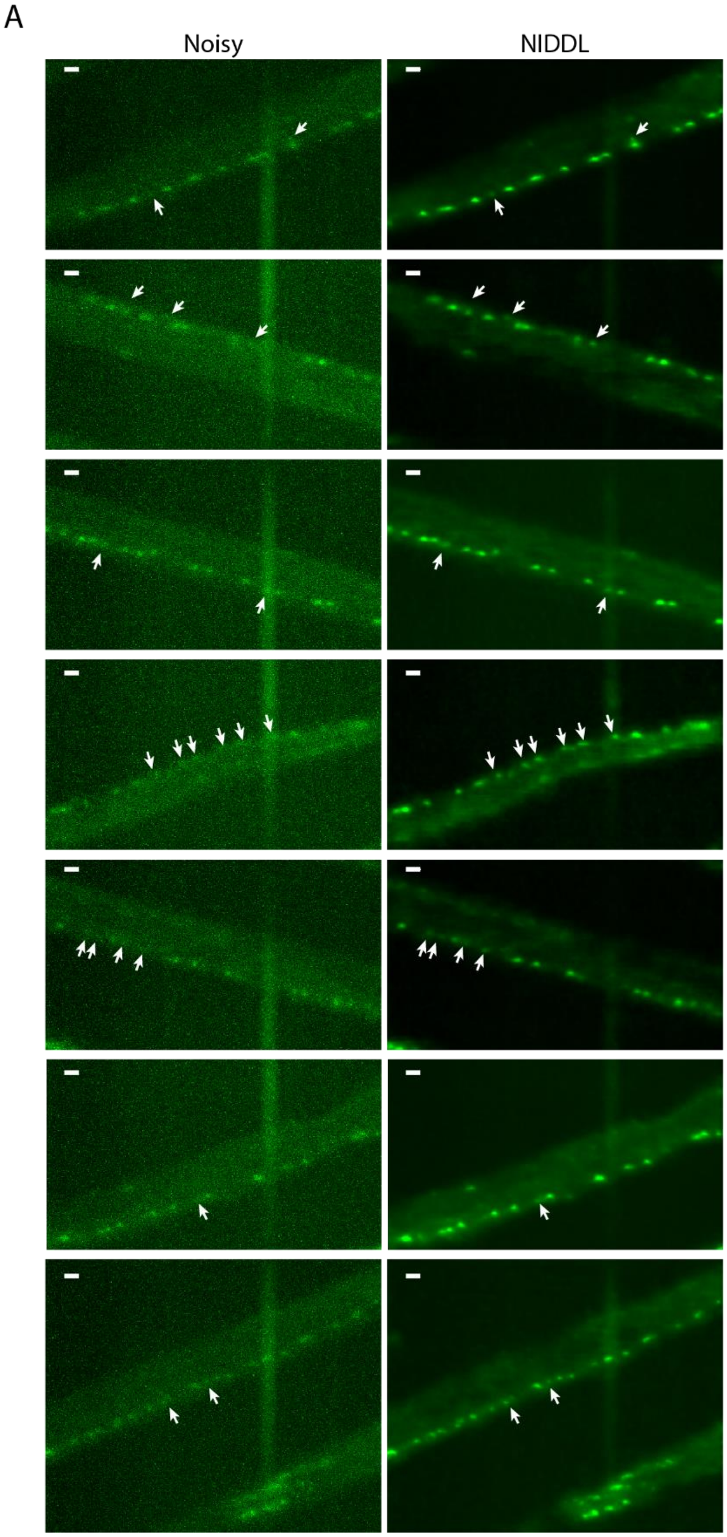

Supplementary Figure 13. Additional examples of deep denoising of large field of view (FOV) noisy image stacks. Left - max projections of noisy images (acquired at low laser power, 20X magnification) showing motor neurons in ventral nerve cord. Right – corresponding deep denoised outputs. (scale bar - 10 μm). Data come from strain OH16230. Arrows indicate example cells that are difficult to identity in noisy images but can be easily identified in denoised images.

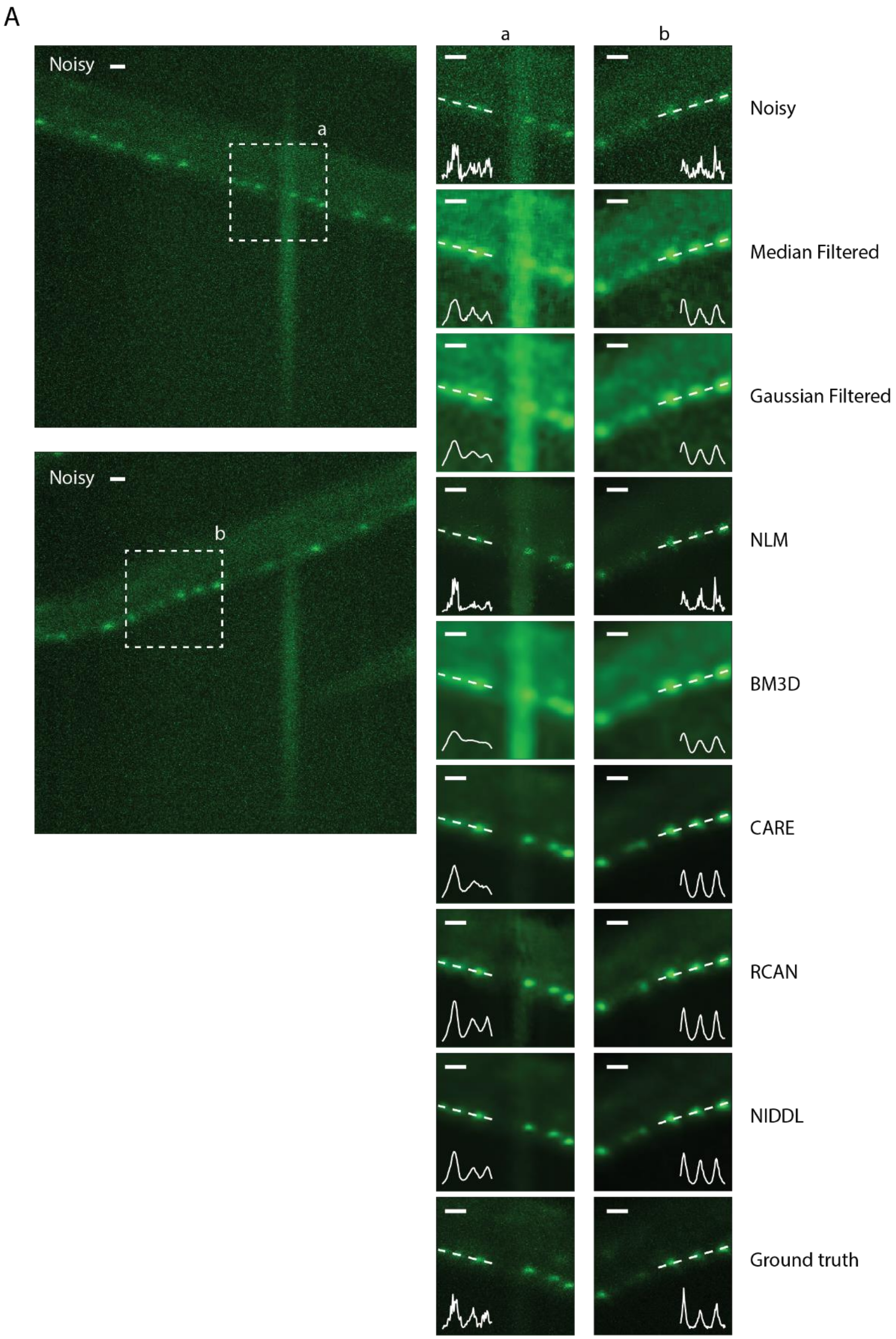

Supplementary Figure 14. Additional qualitative comparison examples of denoising methods on large FOV (20X) motor neuron images. Left - max projections of noisy images (acquired at low laser power, 20X magnification) showing motor neurons in ventral nerve cord. (scale bar - 10 µm). Right – corresponding denoised output generated by different methods shown for dotted box in noisy images. (scale bar - 5 µm). Inset shows intensity profile along the dotted line. Data come from strain OH16230.

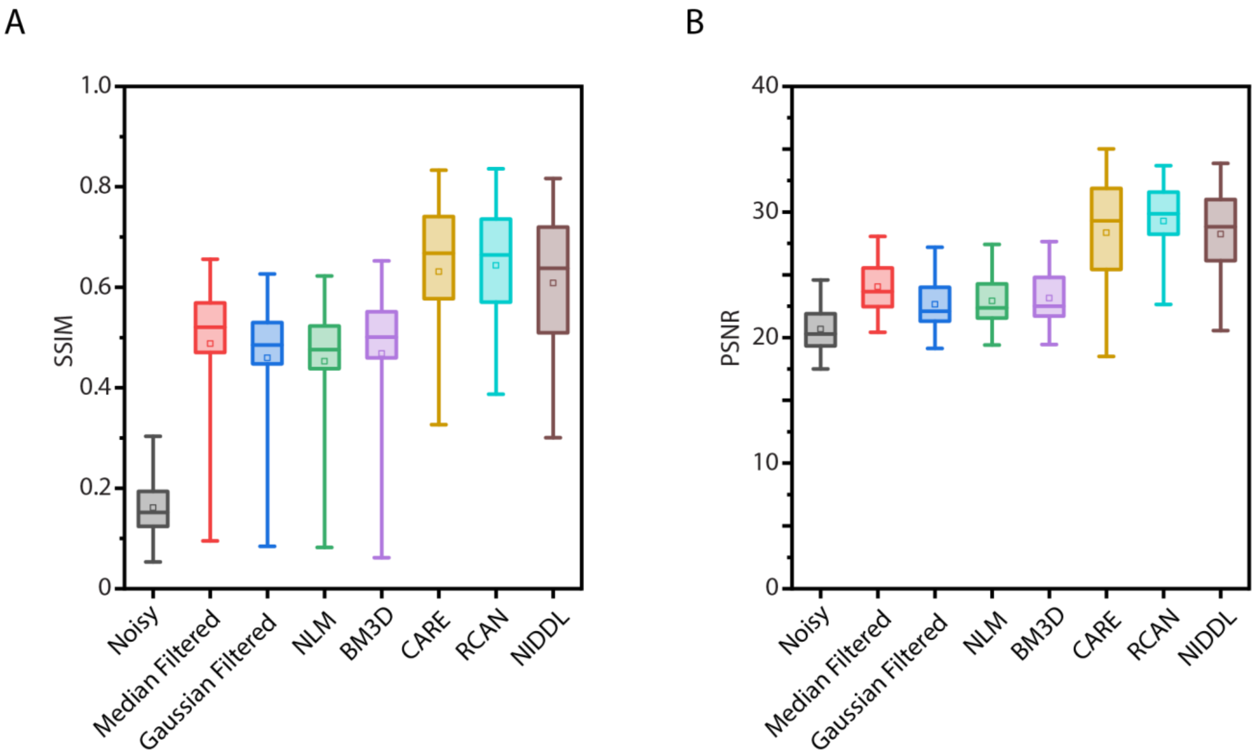

Supplementary Figure 15. Accuracy comparison of NIDDL with traditional denoising methods and deep learning based methods on large FOV (20X magnification) ventral cord data as shown in Fig. 2F. A) SSIM accuracy, B) PSNR comparison across various methods. (n = 60, 60, 60, 60, 60, 60, 73, 217 images across conditions). Box center indicates median, box edges indicate 25th and 75th percentile, and whiskers indicate 5th and 95th percentile. Source data are provided in Source Data file.

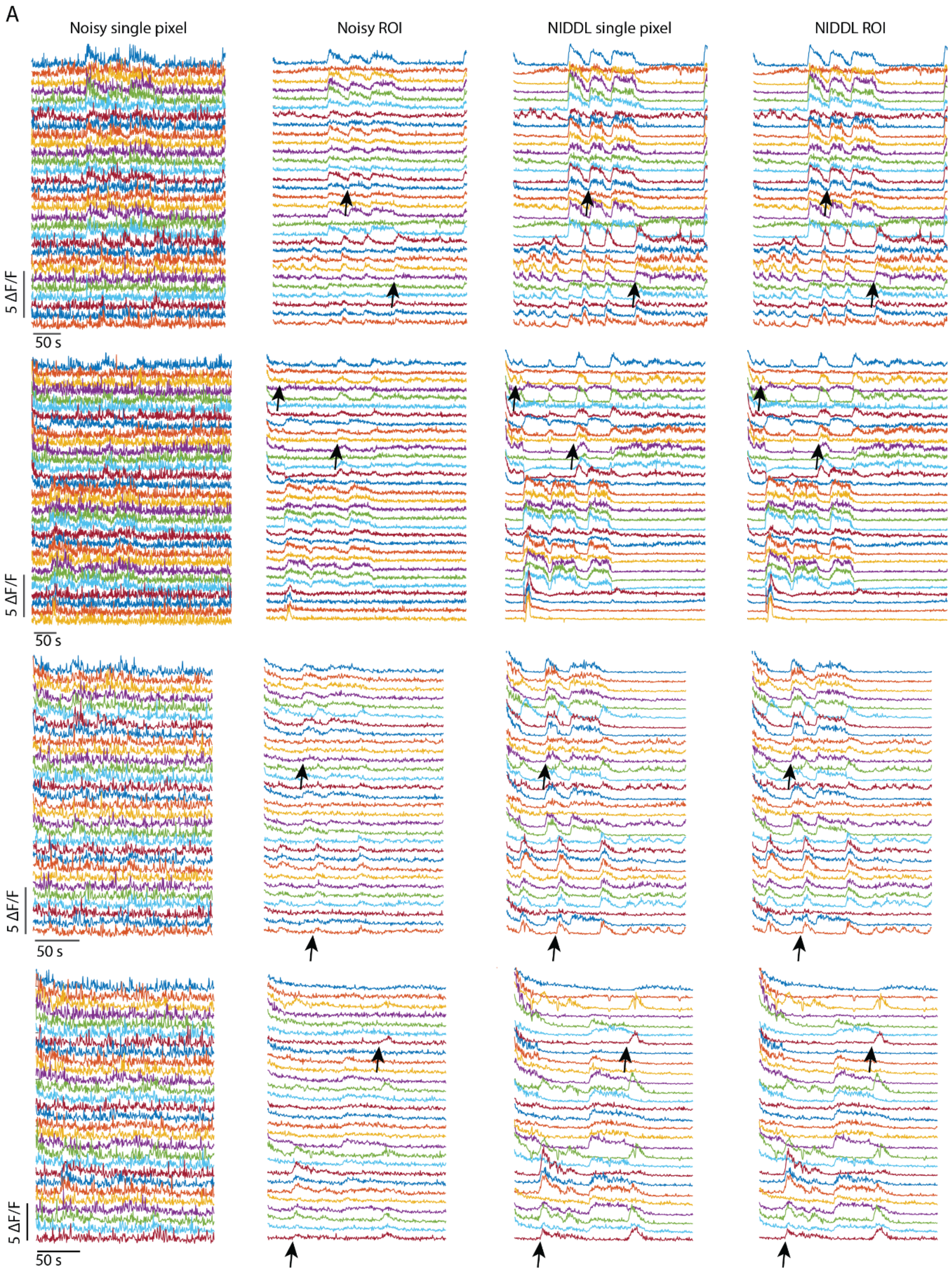

Supplementary Figure 16. Additional examples of ventral cord neurons' activity traces extracted from in-device recordings. A) Four columns in each row show 1) single pixel neuron activity traces from noisy videos, 2) neuron activity traces from noisy videos extracted using ROI mean, 3) single pixel neuron activity traces from denoised video, and 4) neuron activity traces from denoised videos extracted using

ROI mean. Row 1 corresponds to worm 1 in Figure 5C, Row 2 - worm 2, Row 3 - worm 3, Row 4 - worm 4. Arrows indicate examples of neuron activity transients that are lost when traces are extracted by averaging ROI pixels in noisy video. In comparison, single pixel traces or mean ROI traces extracted from denoising video recover such transients.

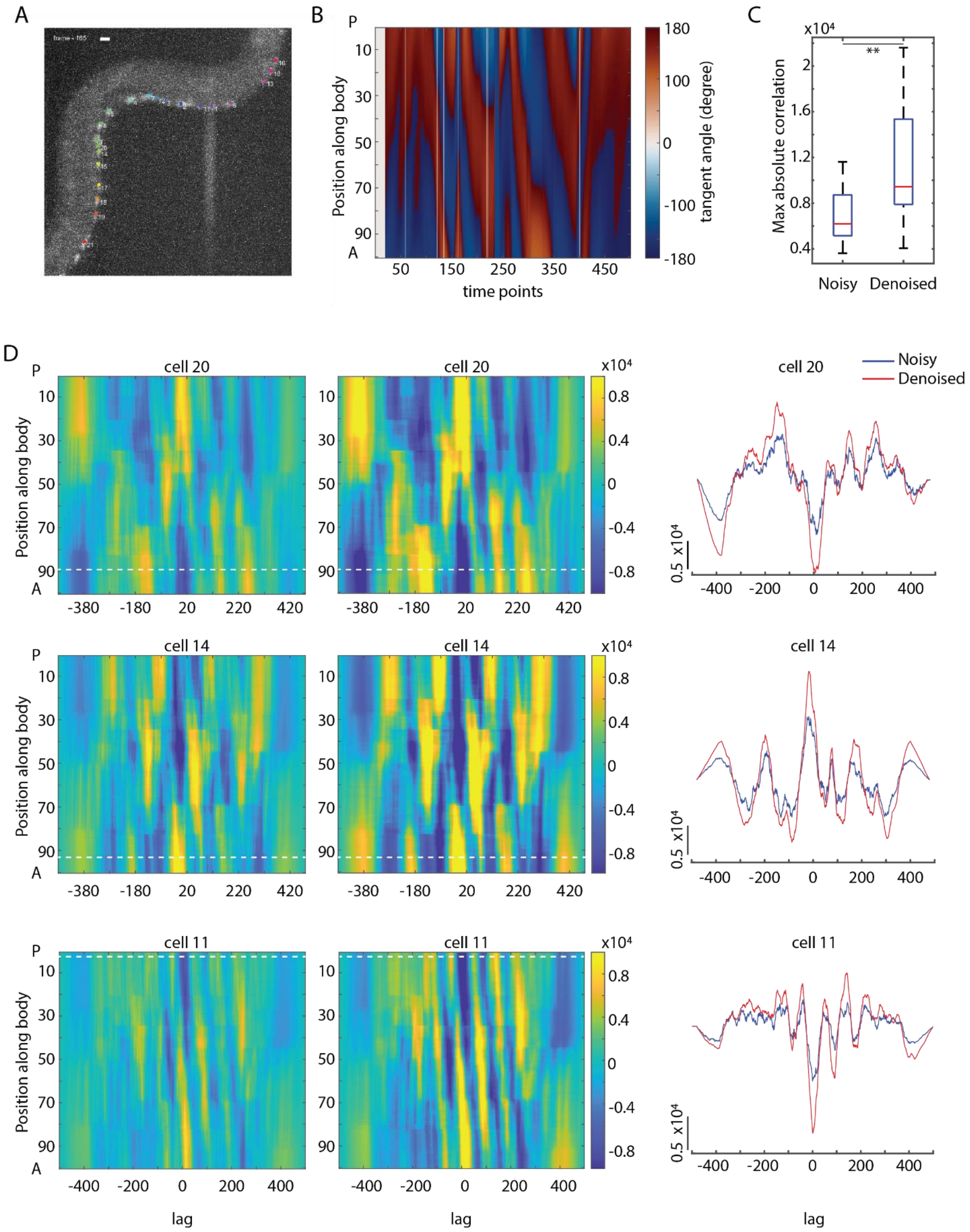

Supplementary Figure 17. Deep denoising enables correlating neuron activity to behavior in freely moving animal. A) Example frame from video recording of freely-moving C. elegans showing tracked ventral cord neurons. (scale bar - 10 µm). B) Tangent angle along the body as it changes with time. 'A' denotes

anterior side and ‘P’ denotes posterior side. C) Comparison of maximum absolute value of correlation between neuron activities and curvature (n = 15 cells, **p<0.01, two-sided paired comparison test). Box centerlines indicate median, box edges indicate 25th and 75th percentile, whiskers indicate most extreme data points D) Left and middle panels – heat map plots of cross correlation of neuron activities to animal curvature. Body portion was discretized into 100 segments. Rows in each heat map indicate cross correlation of neuron activity to curvature at the specific body segment. ‘A’ denotes anterior side and ‘P’ denotes posterior side. White dotted line indicates the position of the cell on the body portion. Right panels – cross correlation along the dotted white line in left and middle panels i.e. cross correlation of neuron activity to local curvature of the body where the cell is located.

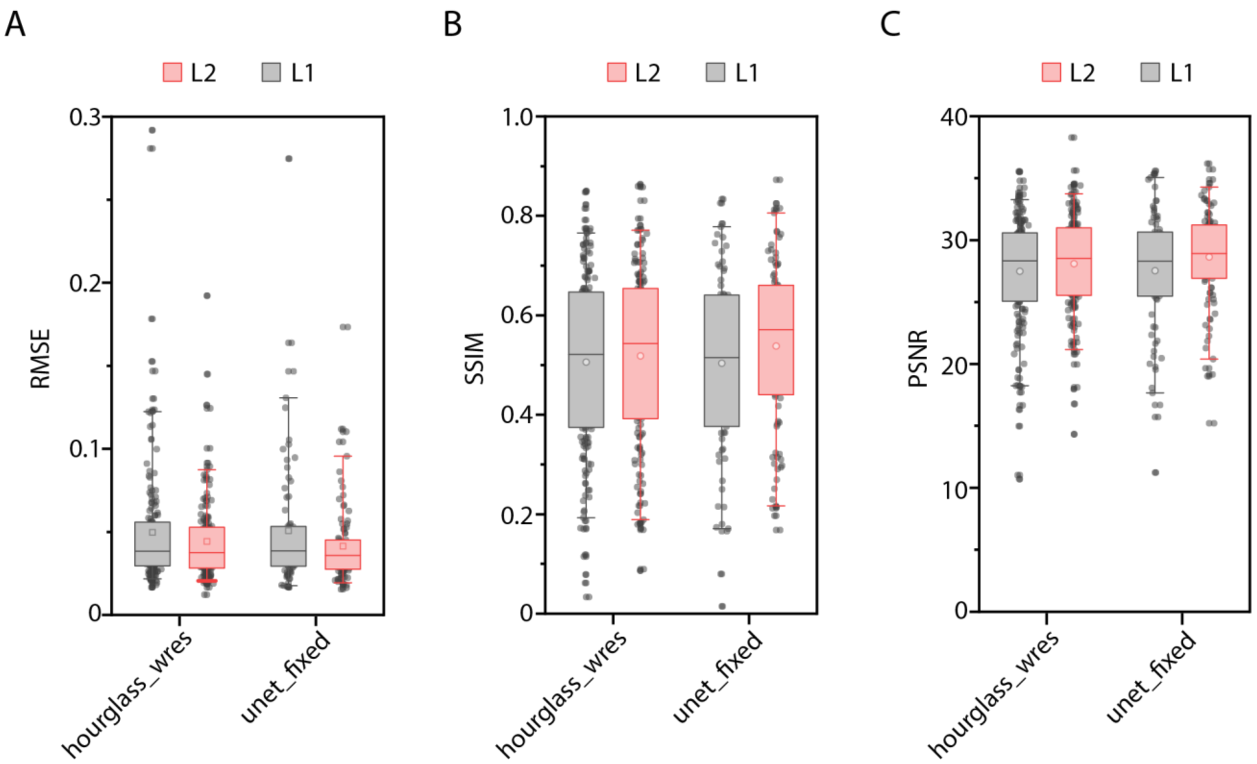

Supplementary Figure 18. Network performance comparisons across two different architectures and loss functions for denoising neurite images. A) RMSE accuracy, B) SSIM accuracy and C) PSNR of deep denoised images from test data set across optimized neural network architectures (‘unet_fixed’ and ‘hourglass_wres’) and loss functions (L1 and L2). Data come from strains GT372 and GT366. (n = 174, 159, 79, 99 images for 4 conditions). Box center indicates median, box edges indicate 25th and 75th percentile, and whiskers indicate 5th and 95th percentile. Source data are provided in Source Data file.

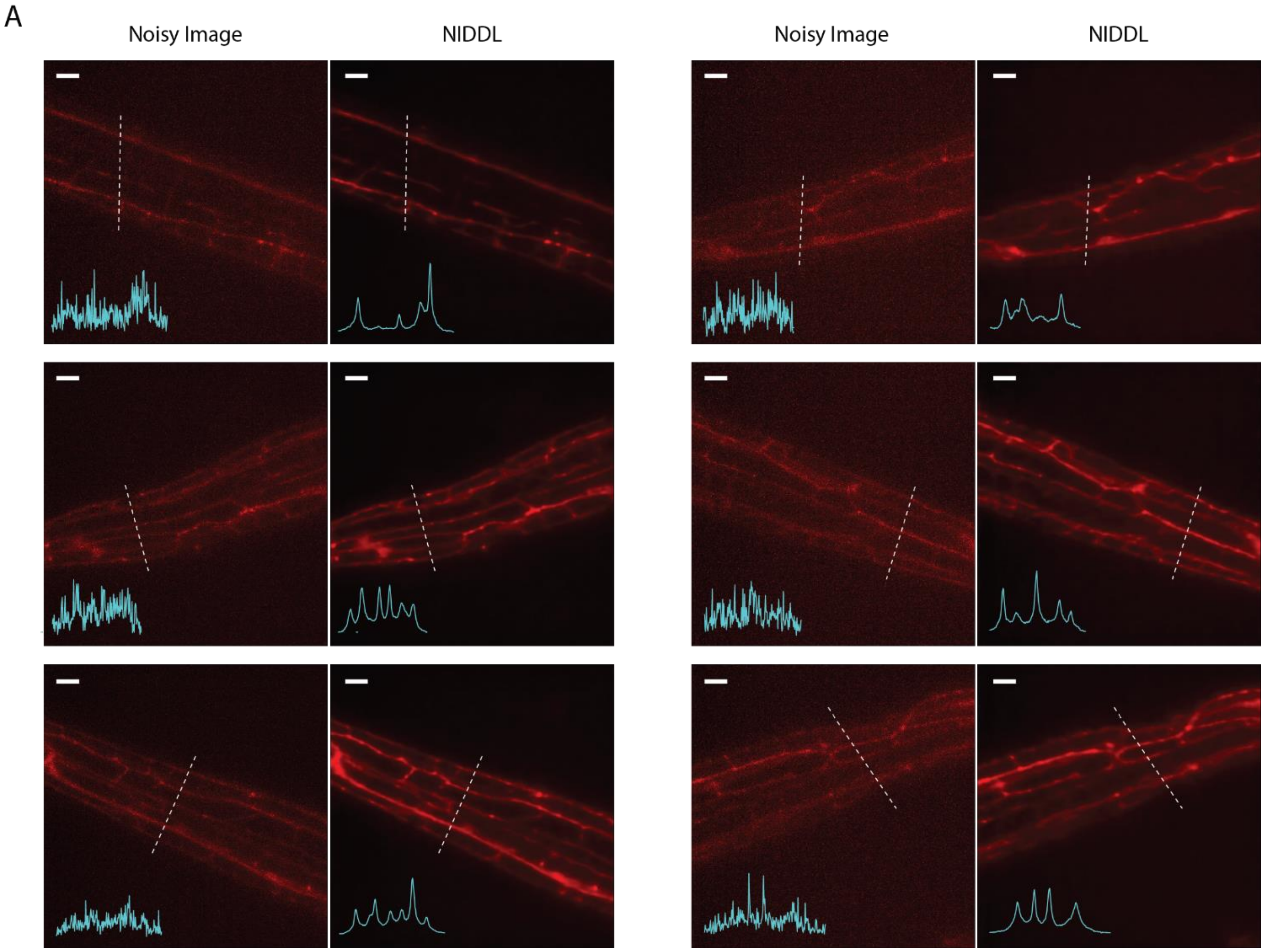

Supplementary Figure 19. Additional examples of deep denoising of harsh touch mechanosensory neuron PVD's neurites. A) Left panels – noisy images (acquired at low laser power), right panels – corresponding deep denoised output. (scale bar - 10 μm). Cyan traces in inset indicate pixel intensities along dotted lines. Data come from strain GT366.

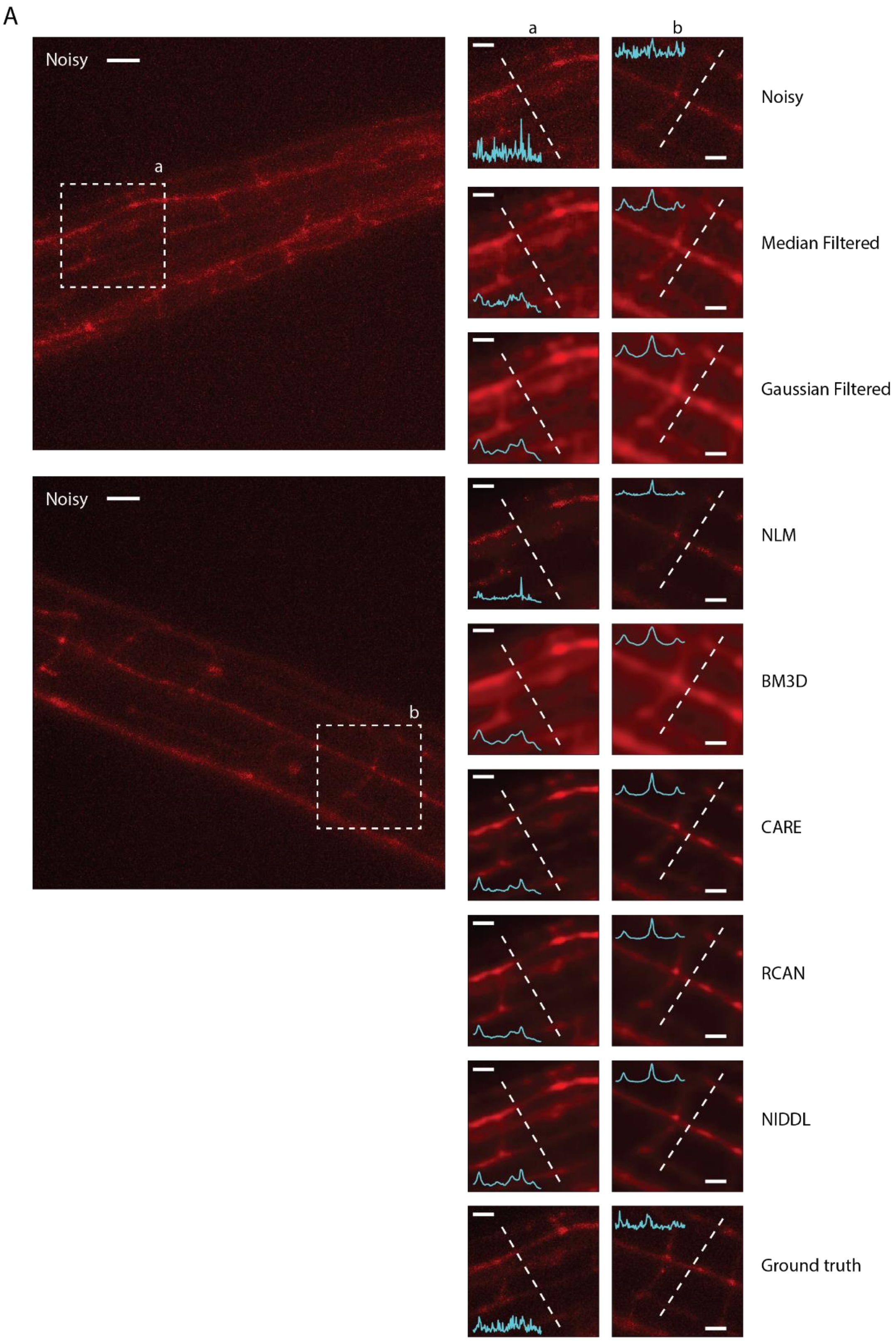

Supplementary Figure 20. Additional qualitative comparison examples of different denoising methods on harsh touch mechanosensory neuron PVD's neurites. Left - max projections of noisy images (acquired at low laser power, 20X magnification) showing motor neurons in ventral nerve cord. (scale bar - 10 μm).

Right – corresponding denoised output generated by different methods shown for dotted box in noisy images. (scale bar - 5 μm). Inset shows intensity profile along the dotted line. Data come from strain OH16230.

A

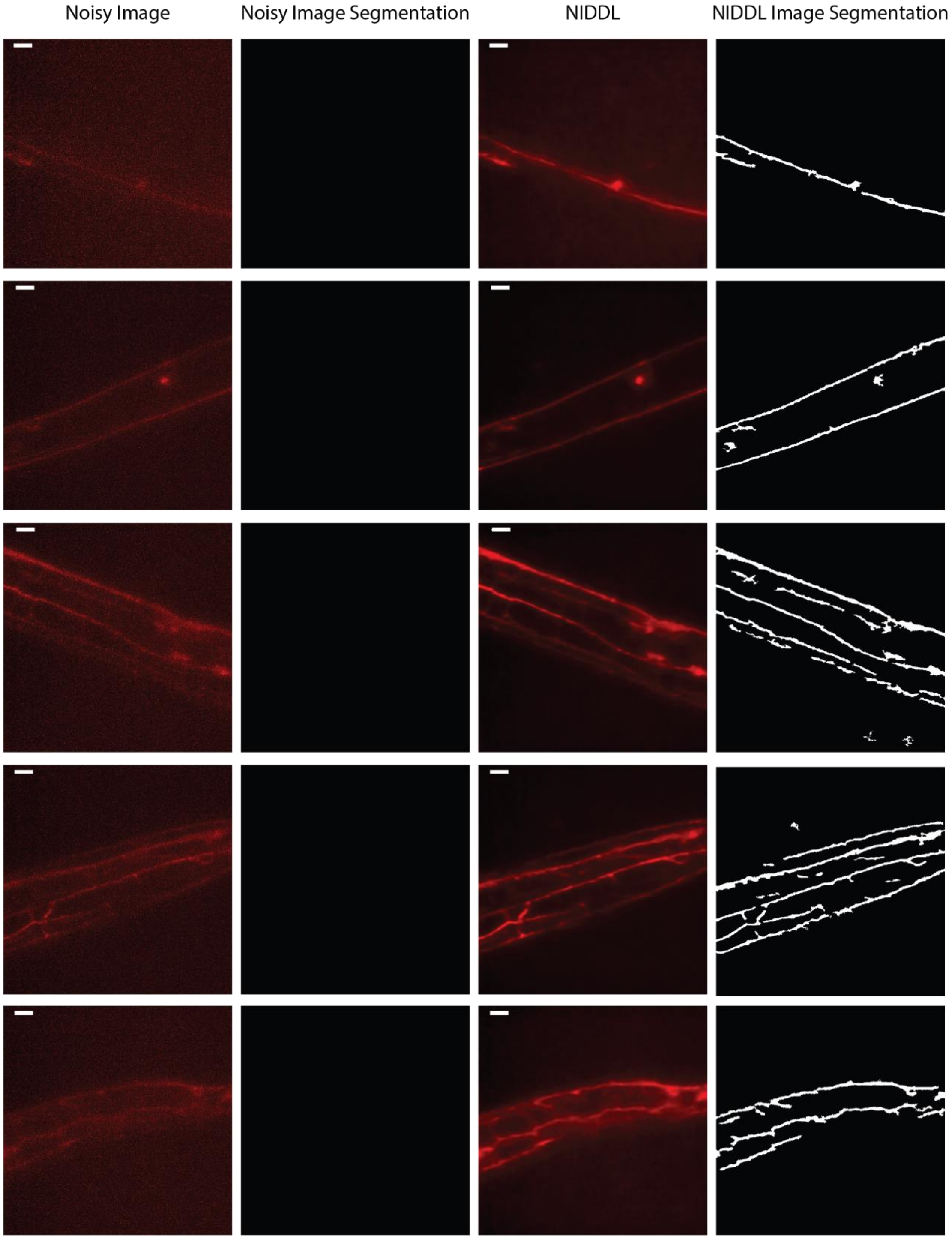

Supplementary Figure 21. Deep denoising of images facilitate neurite segmentation. A) Examples of noisy images showing harsh touch mechanosensory neuron PVD's neurites, no regions are detected in noisy images with simple morphological operations (see Methods – Neurite segmentation), corresponding deep denoised outputs and segmented neurites in denoised images. (scale bar - 10 μm). Data come from strain GT366.

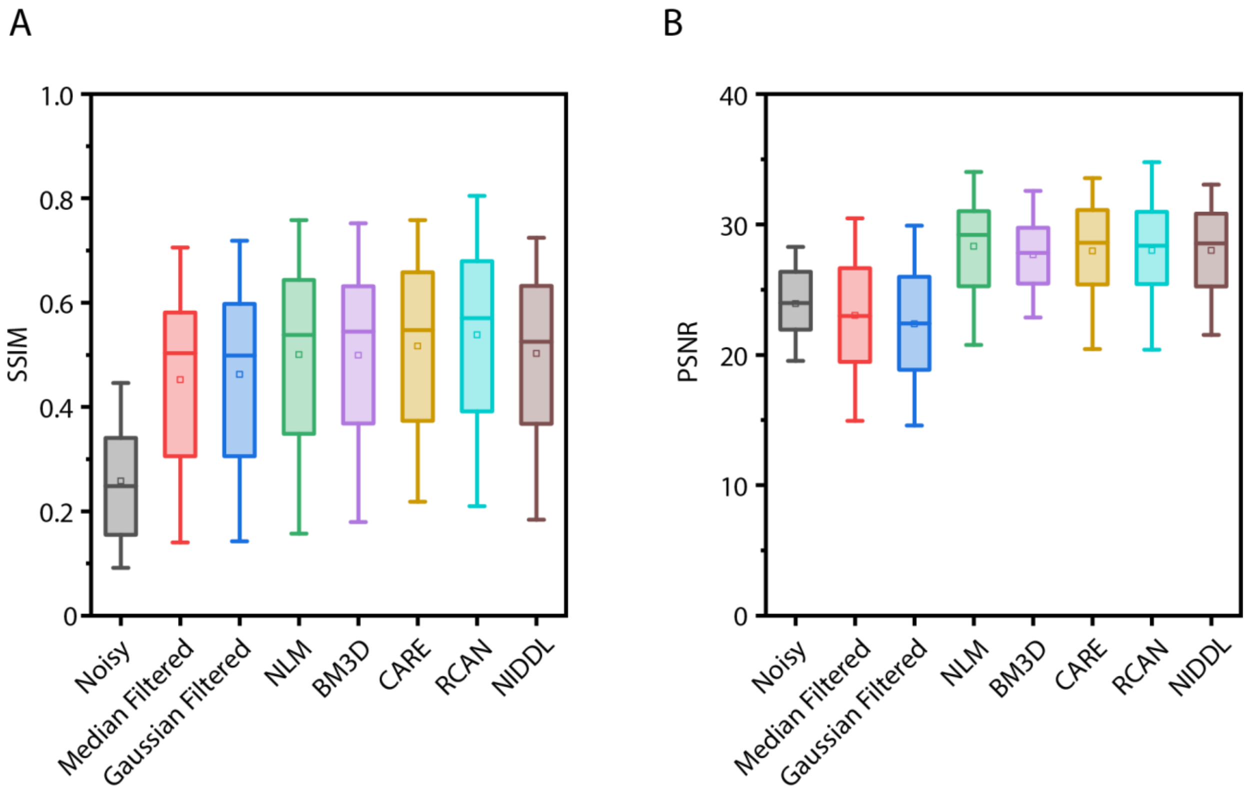

Supplementary Figure 22. Additional accuracy comparison of NIDDL with other non-deep-learning and deep-learning based methods for neurites of gentle touch and harsh touch neurons. A) SSIM accuracy and B) PSNR of noisy images, median filtered images, Gaussian filtered images, and deep denoised images. Data come from strains GT372 and GT366. (n = 86, 86, 86, 86, 86, 86, 443, 443 images across conditions). Box center indicates median, box edges indicate 25th and 75th percentile, and whiskers indicate 5th and 95th percentile. Source data are provided in Source Data file.

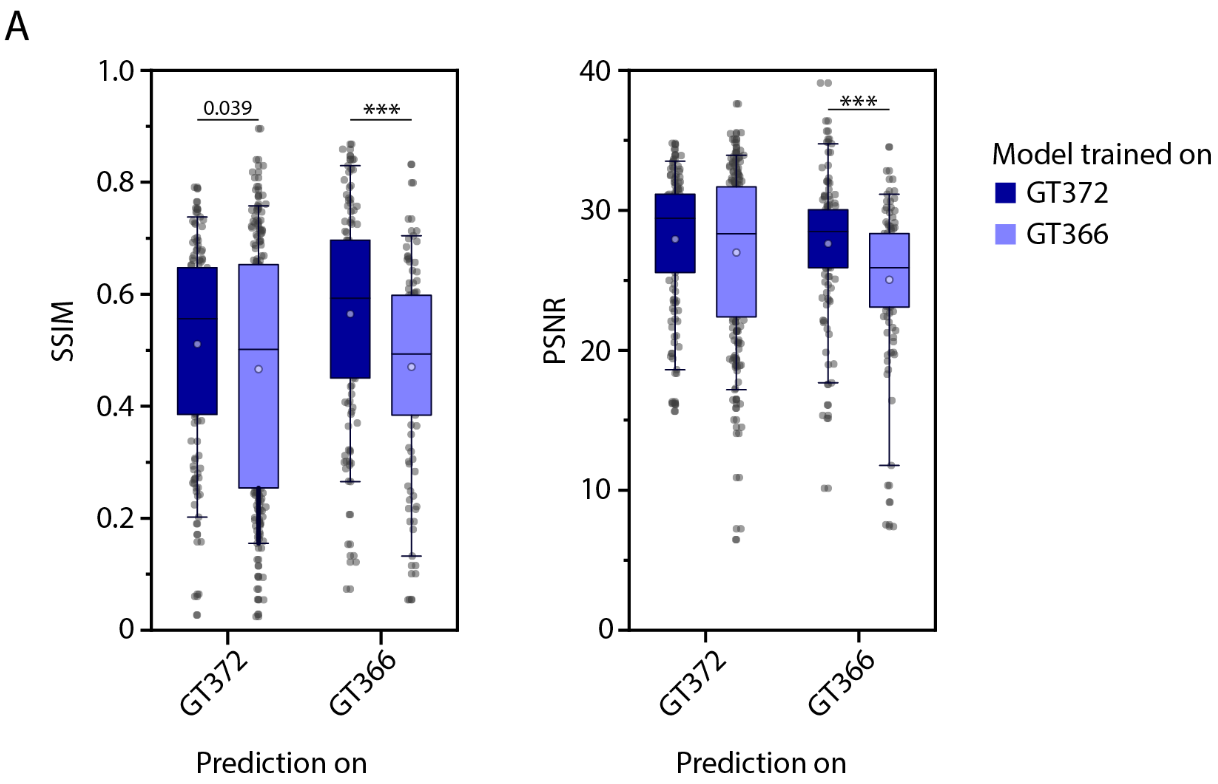

Supplementary Figure 23. Accuracy comparison on noisy images from 2 different strains, GT372 and GT366 that label neurites of gentle touch and harsh touch mechanosensory neurons respectively, when models are trained on specific strain's data. A) Left – SSIM accuracy and right – PSNR achieved by deep denoising when networks are trained on specific strain's data. (n = 129, 203, 118, 97 images for 4 conditions, ***p<0.001, two-sided Holm-Bonferroni paired comparison test). Box center indicates median, box edges indicate 25th and 75th percentile, and whiskers indicate 5th and 95th percentile. Source data are provided in Source Data file.
