# Supplementary material for: Fast, efficient, and accurate neuro-imaging denoising via supervised deep learning
Source: Nat Commun. 2022 Sep 2;13:5165. doi: 10.1038/s41467-022-32886-w (PMC9440141; doi:10.1038/s41467-022-32886-w)
Supplement: Supplementary file 3 — Description of Additional Supplementary Files [file 41467_2022_32886_MOESM3_ESM.pdf]

Description of Additional Supplementary Files

Supplementary Movie 1 – Deep denoising examples of low SNR whole-brain images. Video shows 3 low SNR images from different animals (1 z-plane from 3D stack) acquired at low laser power and corresponding deep denoised image generated by trained network. Data comes from strain ZIM504 and OH16230. Scale bar corresponds to 5 µm.

Supplementary Movie 2 – Deep denoising of whole-brain recording. Max-projection images for low SNR video acquired at low laser power, high SNR video acquired at high laser power, and corresponding deep denoised video generated by network. Cell nuclei are labelled with nuclear localized GCaMP5K. Data comes from strain ZIM504. Scale bar corresponds to 5 µm.

Supplementary Movie 3 – Deep denoising of ventral cord neurons for animals restrained in microfluidic device. Max projection images of low SNR video acquired at low laser power and corresponding deep denoised video. Video shows two animals in microfluidic device. Data comes from strain OH16230. Scale bar corresponds to 10.4 µm.

Supplementary Movie 4 – Deep denoising of ventral cord neurons for freely moving animal. Max projection images of low SNR video acquired at low laser power and corresponding deep denoised video. Data comes from strain OH16230. Scale bar corresponds to 10.4 µm.

Supplementary Movie 5 – Deep denoising of neurites of harsh and gentle touch mechanosensory neurons. Video shows 3 low SNR images (max-projections of 3D stack) from different animals acquired at low laser power and corresponding deep denoised image generated by trained network. Data comes from strain GT372 and GT366. Scale bar corresponds to 5 µm.
