# Supplementary material for: Fast, efficient, and accurate neuro-imaging denoising via supervised deep learning
Source: Nat Commun. 2022 Sep 2;13:5165. doi: 10.1038/s41467-022-32886-w (PMC9440141; doi:10.1038/s41467-022-32886-w)
Supplement: Supplementary file 9 — Reporting Summary [file 41467_2022_32886_MOESM9_ESM.pdf]

nature portfolio

Corresponding author(s): Hang Lu

Last updated by author(s): Jul 8, 2022

Reporting Summary

Nature Portfolio wishes to improve the reproducibility of the work that we publish. This form provides structure for consistency and transparency in reporting. For further information on Nature Portfolio policies, see our Editorial Policies and the Editorial Policy Checklist.

Statistics

For all statistical analyses, confirm that the following items are present in the figure legend, table legend, main text, or Methods section.

n/a	Confirmed	
☐	☒	The exact sample size (n) for each experimental group/condition, given as a discrete number and unit of measurement
☐	☒	A statement on whether measurements were taken from distinct samples or whether the same sample was measured repeatedly
☐	☒	The statistical test(s) used AND whether they are one- or two-sided<br>*Only common tests should be described solely by name; describe more complex techniques in the Methods section.*
☒	☐	A description of all covariates tested
☐	☒	A description of any assumptions or corrections, such as tests of normality and adjustment for multiple comparisons
☐	☒	A full description of the statistical parameters including central tendency (e.g. means) or other basic estimates (e.g. regression coefficient) AND variation (e.g. standard deviation) or associated estimates of uncertainty (e.g. confidence intervals)
☐	☒	For null hypothesis testing, the test statistic (e.g. F, t, r) with confidence intervals, effect sizes, degrees of freedom and P value noted<br>*Give P values as exact values whenever suitable.*
☒	☐	For Bayesian analysis, information on the choice of priors and Markov chain Monte Carlo settings
☒	☐	For hierarchical and complex designs, identification of the appropriate level for tests and full reporting of outcomes
☐	☒	Estimates of effect sizes (e.g. Cohen's d, Pearson's r), indicating how they were calculated

Our web collection on statistics for biologists contains articles on many of the points above.

Software and code

Policy information about availability of computer code

Data collection	Data was collected using Bruker Opterra II Swept field confocal microscope system. Commercial software (Prairie View) for the microscope system was used for acquisition of the data.
Data analysis	Custom codes (https://github.com/shiveshc/whole-brain_DeepDenoising) developed in MATLAB R2020b and Python 3.5 were used. For Python codes; several open-source libraries were used as described in "requirements.txt file" along with code submission.

For manuscripts utilizing custom algorithms or software that are central to the research but not yet described in published literature, software must be made available to editors and reviewers. We strongly encourage code deposition in a community repository (e.g. GitHub). See the Nature Portfolio guidelines for submitting code & software for further information.

Data

Policy information about availability of data

All manuscripts must include a data availability statement. This statement should provide the following information, where applicable:

- Accession codes, unique identifiers, or web links for publicly available datasets
- A description of any restrictions on data availability
- For clinical datasets or third party data, please ensure that the statement adheres to our policy

Sample datasets to run trained networks are available at https://github.com/shiveshc/whole-brain_DeepDenoising. Raw imaging datasets used to train networks and various calcium imaging datasets will be available upon request due to the large size. The data generated in this study are provided in the Source Data file. OpenWorm atlas was obtained from https://doi.org/10.1098/rstb.2017.0382.

Field-specific reporting

Please select the one below that is the best fit for your research. If you are not sure, read the appropriate sections before making your selection.

- [x] Life sciences
- [ ] Behavioural & social sciences
- [ ] Ecological, evolutionary & environmental sciences

For a reference copy of the document with all sections, see nature.com/documents/nr-reporting-summary-flat.pdf

Life sciences study design

All studies must disclose on these points even when the disclosure is negative.

Sample size	Training dataset sample size was determined based on accuracy comparisons of number of images needed by neural network model to achieve maximum accuracy (Fig. 1D, Supp Fig. 6). Test dataset sample size across all comparisons (whole-brain imaging, ventral cord neurons imaging, mechanosensory neurite imaging) ranges from 40 images to ~2,000 images. We used n-fold validation so the test data sets are the held-out data.
Data exclusions	No data were excluded.
Replication	Accuracy was confirmed using test datasets completely held out from network training. For all applications and comparisons, 10 instances of networks were trained with a randomly selected subset of data each time. The accuracy was shown to replicate.
Randomization	To compare accuracy across network architectures, loss functions, and training modes, several instances of networks were trained using randomly sampled subsets of data in each run. Further, accuracy was quantified using held out test datasets.
Blinding	Blinding in acquisition is not possible because the samples have to be manually imaged; blinding is also not necessary as images need to have identity to act as training or testing data. In analysis, selection of datasets were randomized by computer and thus blinded.

Reporting for specific materials, systems and methods

We require information from authors about some types of materials, experimental systems and methods used in many studies. Here, indicate whether each material, system or method listed is relevant to your study. If you are not sure if a list item applies to your research, read the appropriate section before selecting a response.

Materials & experimental systems

n/a	Involved in the study	
☒	☐	Antibodies
☒	☐	Eukaryotic cell lines
☒	☐	Palaeontology and archaeology
☐	☒	Animals and other organisms
☒	☐	Human research participants
☒	☐	Clinical data
☒	☐	Dual use research of concern

Methods

n/a	Involved in the study	
☒	☐	ChIP-seq
☒	☐	Flow cytometry
☒	☐	MRI-based neuroimaging

Animals and other organisms

Policy information about studies involving animals; ARRIVE guidelines recommended for reporting animal research

Laboratory animals	For all experiments, C. elegans were cultured using standard techniques. A detailed list of strains used in this work is provided in the manuscript. All data were collected on larval stage 4 C. elegans hermaphrodites.
Wild animals	No wild animals were used in the study.
Field-collected samples	No field-collected samples were used in the study.
Ethics oversight	The study did not require an ethical approval.

Note that full information on the approval of the study protocol must also be provided in the manuscript.
